# The full-ORF clone resource of the German cDNA Consortium

**DOI:** 10.1186/1471-2164-8-399

**Published:** 2007-10-31

**Authors:** Stephanie Bechtel, Heiko Rosenfelder, Anny Duda, Christian Peter Schmidt, Ute Ernst, Ruth Wellenreuther, Alexander Mehrle, Claudia Schuster, Andre Bahr, Helmut Blöcker, Dagmar Heubner, Andreas Hoerlein, Guenter Michel, Holger Wedler, Karl Köhrer, Birgit Ottenwälder, Annemarie Poustka, Stefan Wiemann, Ingo Schupp

**Affiliations:** 1 Department of Molecular Genome Analysis, German Cancer Research Center (DKFZ), Heidelberg, Germany; 2 Qiagen GmbH, Hilden, Germany; 3 Department of Genome Analysis, Helmholtz Centre for Infection Research (HZI), Braunschweig, Germany; 4 AGOWA GmbH, Berlin, Germany; 5 RZPD GmbH, Heidelberg, Germany; 6 University of Duesseldorf, Duesseldorf, Germany; 7 Eurofins Medigenomix GmbH, Martinsried, Germany

## Abstract

**Background:**

With the completion of the human genome sequence the functional analysis and characterization of the encoded proteins has become the next urging challenge in the post-genome era. The lack of comprehensive ORFeome resources has thus far hampered systematic applications by protein gain-of-function analysis. Gene and ORF coverage with full-length ORF clones thus needs to be extended. In combination with a unique and versatile cloning system, these will provide the tools for genome-wide systematic functional analyses, to achieve a deeper insight into complex biological processes.

**Results:**

Here we describe the generation of a full-ORF clone resource of human genes applying the Gateway cloning technology (Invitrogen). A pipeline for efficient cloning and sequencing was developed and a sample tracking database was implemented to streamline the clone production process targeting more than 2,200 different ORFs. In addition, a robust cloning strategy was established, permitting the simultaneous generation of two clone variants that contain a particular ORF with as well as without a stop codon by the implementation of only one additional working step into the cloning procedure. Up to 92 % of the targeted ORFs were successfully amplified by PCR and more than 93 % of the amplicons successfully cloned.

**Conclusion:**

The German cDNA Consortium ORFeome resource currently consists of more than 3,800 sequence-verified entry clones representing ORFs, cloned with and without stop codon, for about 1,700 different gene loci. 177 splice variants were cloned representing 121 of these genes. The entry clones have been used to generate over 5,000 different expression constructs, providing the basis for functional profiling applications. As a member of the recently formed international ORFeome collaboration we substantially contribute to generating and providing a whole genome human ORFeome collection in a unique cloning system that is made freely available in the community.

## Background

Recent efforts have completely unravelled also the human genome sequence [1-6]. Since, attention has shifted towards the detailed understanding of gene functions in health and disease by analysing the structure, biological activities and dynamics of the encoded proteins. To this end, RNA interference (RNAi) has received much attention as a powerful tool for systematic loss-of-function genetic studies on a large scale [7-9]. However, for many functional genomics and proteomics applications including studies on protein subcellular localization [10], protein structures [11,12], protein functions in cell-based experiments [13,14], analysis of protein-protein interactions [15,16], and disease-related processes [17,18], expression clones are indispensable. The clones of cDNA collections [2,5,6,19] are generally not ideal for immediate use in these experiments, as they contain 5'and 3'untranslated regions (UTRs) of varying lengths. These interfere with the expression of the encoded proteins especially when coexpression of in-frame fusions with specific tags at either ends are anticipated. The 5'UTRs may contain in-frame stop codons or lead to the inclusion of artificial amino acid sequences. The native stop codon that terminates any ORF furthermore impedes the expression of C-terminal protein fusions. In consequence, the generation of clone collections that only contain the protein coding part of the genes (ORFs) has become a key component for the comprehensive and systematic analysis of protein functions in many different systems. Despite the availability of the human genome sequence, a respective full-ORF clone collection is far from being complete [20]. This is in part due to the fact that the structures of many genes are still unclear, and thus require considerable manual and individual verification [21]. Furthermore, the phenomenon of alternative splicing has not received much attention in ORF clone collections yet. Here, we report on the production of a full-length ORF clone library of human genes and splice forms, using the recombination-based Gateway cloning system (Invitrogen) [22]. We have developed a cloning approach applied to more than 2,200 different ORFs including (1) optimization and improvement of gene models, and of the ORF amplification and cloning processes, (2) development of a cloning strategy to simultaneously generate Gateway entry clones with and without stop codon, (3) establishment of a pipeline for ORF sequence validation (4) programming and implementation of a sample tracking database. The generated entry clone resource currently comprises more than 3,800 sequence-validated Gateway clones for more than 1,850 ORFs, the coding sequences have an average size of greater 2 kb. As a member of the recently initiated international ORFeome collaboration [20] we significantly contribute to generating and providing ORF clone resources for all human genes and their splice forms in a unique and flexible cloning system. The Gateway entry clones have since been used to generate over 5,000 different expression constructs that have been successfully exploited in functional profiling applications [13,14,23,24]. All entry clones are available through the international ORFeome collaboration [25].

## Results and Discussion

### Gene structures and models

**Figure 1:**
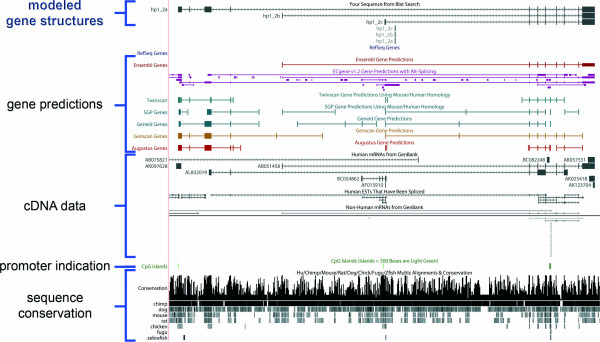
**Modeling of gene structures**. Screen shot of the UCSC Genome browser displaying a gene that we predicted to be expressed in three variant transcripts and that is consequently characterized by three gene models (hp1_2a-c). The gene models show different transcription start sites, resulting in different N-terminal ends of the encoded proteins. All three models could be verified by ORF amplification, cloning, and sequence validation of the resulting entry clones.

A number of automated software solutions have been implemented to annotate genomes and genes [26,27]. Also then the quality of gene predictions is tightly associated with the availability and quality of cDNA sequence information as most gene predictions are based on cDNA sequences [27,28]. Nevertheless, automated gene predictions are not perfect, and careful manual annotation is thus the method of choice in gene structure modeling [29]. We systematically performed manual annotation of genes and gene structures using available sequence information from mammalian species and computational gene predictions. The combined data was used to create gene models and virtual templates, to finally predict functional ORFs for subsequent cloning and sequence validation. The German cDNA Consortium focuses on the ORF cloning of genes not yet present in ORF clone collections. Thus far, some 2,500 gene loci have been annotated, identifying more than 2,200 full-ORF variants of about 2,000 genes. cDNAs, either annotated as mRNA or ESTs, were available for more than 1,850 genes (either DKFZ or MGC clones), while RT-PCR amplification was required for about 150 gene and ORF-models. These covered mostly long and lowly expressed genes. Figure 1 shows an example of a gene model for which three alternative transcript starts were predicted in our annotation, all of which have since been confirmed by sequencing of cloned RT-PCR products. cDNAs were not available to amplify the ORFs of that gene.

### Efficient ORF amplification procedure

#### - Tagging the ORFs with Gateway sites

**Figure 2:**
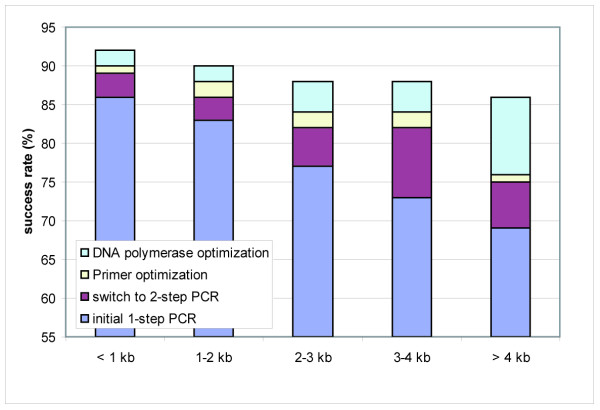
**PCR-success with and without optimization of the reaction conditions**. The impact of the optimizations steps on the success rates (in percentages) are shown in dependence on the ORF size. A PCR was defined successful when a DNA product of the expected size was observed in analytical agarose gel electrophoresis.

For systematic cloning of the ORFs, the recombination-based Gateway technology (Invitrogen) was used [22] as it allows their functional exploitation in a broad range of expression systems in parallel. Initially, the ORFs had been amplified by a one-step PCR [10]. Many ORFs then failed to be amplified due to the length of the primer and the average success rate was < 75 % (Fig. 2). This effect increased with increasing ORF size, resulting in a > 30 % PCR failure rate for ORFs > 3 kb (Fig. 2). Further, the error rate within the primer sites was unacceptably high in cloned products because of the difficulty to reliably generate long oligonucleotides. By the switch to a 2-step PCR strategy described in [30], according to [31,32] a clear increase in the PCR success rate of up to 15 % could be achieved, especially for ORFs > 3 kb (Fig. 2). In addition, this strategy permitted the use of only one universal primer set suitable for all second step PCRs, resulting in a reduction of costs for the shortened ORF-specific primers. Detailed protocols on this and the other procedures in the cloning process are available at [33].

#### - Primer quality and processivity and fidelity of DNA polymerases

Although the primer truncation strongly reduced the PCR failure rate, we still observed primer errors when sequencing the ORF clones rendering the affected clones useless. The error rate could be further reduced by selecting the oligonucleotides with the highest sequence accuracy identified in a comparative test of three suppliers (Table 1). Whereas for two suppliers mostly frame-shift mutations caused by a high rate of n-1 primers, were observed, only 3 % of primers from a third supplier were incorrect (Table 1). It should be noted, however, that the quality of suppliers is variable and that care should be taken to follow the success rate over time.

**Table 1:** Comparison of primer quality of three different suppliers

	**total # of analysed clones**	**% of clones with frame shift mutations**	**% of clones with missense mutations**	**% of positive clones**
**Supplier 1**	100	8	3	89
**Supplier 2**	100	5	1	94
**Supplier 3**	100	1	2	97

**Figure 3:**
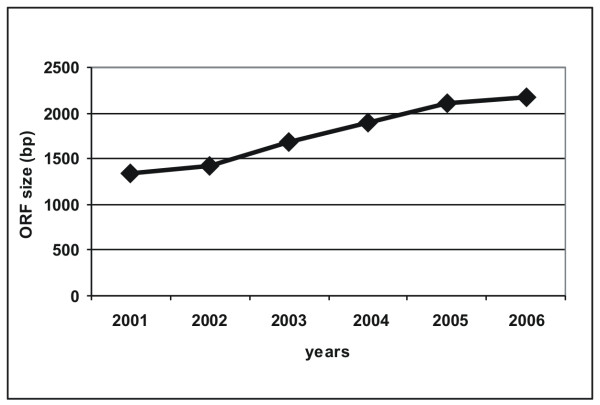
**Average size of ORFs cloned in the project**. The continuous increase in the size of successfully cloned ORFs due to the combined improvements of ORF amplification and cloning procedures is shown with respect to the year.

**Figure 4:**
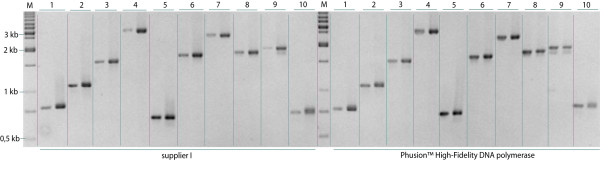
**Comparison of PCR products amplified with two different DNA polymerase systems**. A total of 100 ORFs (50 ORFs per enzyme mix), ranging from 300 to 4,000 bp in size, were amplified. Electrophoretic analysis of 10 representative ORFs amplified using either the supplier I (left panel) or the Phusion™ High-Fidelity DNA Polymerase (Finnzymes) (right panel). One-tenth of each reaction product of first and second step ORF amplification were loaded adjacent to each other on an analytical agarose gel. According to the lane number the expected ORF sizes and accession numbers of first-step PCR templates are as follows: 1: 759 bp, BC100921; 2: 1125 bp, BC093648; 3: 1554 bp, BC104948; 4: 3198 bp, BC117368; 5: 651 bp, BC105131; 6: 1653 bp, BC109061; 7: 2400 bp, BC113416; 8: 1737 bp, BC117320; 9: 1854 bp, BC101755; 10: 720 bp, BC113739.'M' indicates the molecular weight marker lanes.

ORFs with continuously increasing size have been cloned in the course of the project (Fig. 3), now being 2.2 kb on average. In this context, a high PCR failure and mutation rate was observed caused by the DNA polymerase used. We tested two proofreading DNA polymerases mixtures, that had ranked best in a comparative prescreen with ten different enzymes (data not shown), and there the Phusion High-fidelity DNA polymerase (Finnzymes) was identified as the enzyme possessing a high processivity (Fig. 4) but a 30-fold higher fidelity compared to the second enzyme. Hence this enzyme was used for all subsequent PCR reactions. It enhanced the success rate especially of ORFs > 4 kb and ORFs amplified from primary cDNA up to 15 % (Fig. 2) in combination with the addition of DMSO, reported to reduce secondary structures particularly in GC-rich template stretches [34,35] and the reduction of the extension temperature from 72 to 63°C [36].

Where a PCR fragment of expected size could not be obtained, a further round of reamplification was undertaken which was successful in 36 % of these cases (Table 2). The failure rate was especially high when using primary cDNAs (Table 2). This concerned mainly large and lowly and/or only rarely expressed genes and even the pooling of cDNAs from various tissues resulted only in few cases in a fragment of correct size.

**Table 2:** Success rates of ORF amplification

**template type**	**cDNA clone**				**EST clone**				**primary cDNA**				**total**			
**ORFs targeted**	**# total**	**# pos**^a^	**%**^b^	**%**^c^	**# total**	**# pos**^a^	**%**^b^	**%**^c^	**# total**	**# pos**^a^	**%**^b^	**%**^c^	**# total**	**# pos**^a^	**%**^b^	**%**^c^

**1. amplification**	1712	1303	76	76	368	267	73	73	156	39	25	25	2236	1609	72	72
**reamplification**^d^	409	162	40	9	101	39	39	11	117	25	21	16	627	226	36	10
**amplified with alternative template**	103	80	78	5	28	22	79	6	61	15	25	10	192	117	61	5
**amplified with alternative 1-step primers**	32	26	81	2	16	13	81	4	21	6	29	4	69	45	65	2
**Sum**	1712^e^	1571		92	368^e^	341		94	156^e^	85		55	2236^f^	1997		89

^a ^Number of ORFs with expected product size obtained by PCR.

^b ^Sucess rate of the respective reaction step.

^c ^Contribution of each step to the final PCR success rate (rounded values).

^d ^Reamplification with slight modifications of the PCR protocol depending on result of first amplification.

^e ^Sum of ORFs targeted per template type.

^f ^Total sum of ORFs targeted

However, if the amplification was clone-based and the expected PCR product was not obtained, the template DNA was sequence controlled. More than 10 % of all clones used did not contain the expected insert probably due to picking or annotation errors, or they did not contain the complete ORFs. If available, the amplification was repeated with an alternative template which proved to be efficient for ≥ 78 % of these ORFs (Table 2). Where the amplification failed due to no priming or mispriming events, first-step primer redesign generated a PCR fragment in 81 % of the cases (Table 2).

By the application of our PCR pipeline optimized by the combination of amplification step improvements up to 92 % of the ORFs could be successfully amplified (Fig. 2) and more than 86 % irrespective of the ORF size (upper limit tested: 6.5 kb) (Fig. 2; Table 2). We successfully generated amplicons for a total of 1997 different ORFs (Table 2) which were subsequently subjected to BP cloning.

### Recombinatorial cloning of target ORFs

**Figure 5:**
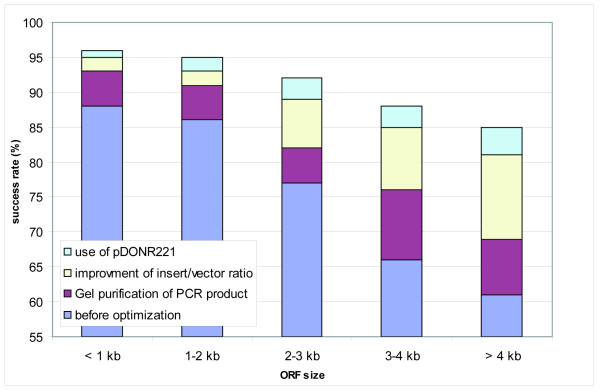
**Success in entry clone production with and without optimization of the reaction compositions and conditions**. The cumulative effect of the different protocol modifications on the BP cloning success is shown for different ORF size ranges. BP reactions were rated successful when the expected ORF could be cloned and sequence verified.

When cloning the ORFs into Gateway entry donor vectors, we identified the DNA purity as a critical parameter in the cloning process. Unspecific side-products, often short contaminations which were particularly observed when the ORFs had been amplified via RT-PCR, recombined during the BP reaction more efficiently than the desired PCR products. This effect increased with larger ORF sizes. The recombination success rate could be improved by more than 15 % by gel-purification of the ORFs, proved to be most advantageous, especially for ORFs > 3 kb compared to ethanol precipitation or even column-purification (Fig. 5).

In addition, the BP cloning was as more efficient as more balanced the molar ratio of PCR product and donor vector was (1:1), or even was shifted in favor of the PCR product. This turned out to be a challenge particularly for low yields of purified PCR products and for ORFs > 2 kb being amplified from primary cDNA, as uneven ratios resulted in only few clones mostly containing only the empty donor vector. Pooling multiple PCR reactions prior to BP cloning improved the success rate by about 20 % (Fig. 5) and was superior to raising the cycle number per reaction as this would have led to increased error rates. The BP cloning efficiency was further enhanced by replacing the Gateway donor vector pDONR201 with the "second-generation" pDONR221 (Fig. 5) reported to improve the recombination efficiency due to modifications of the *att*P1 and *att*P2 site [37]. In total, more than 1,800 amplicons were successfully cloned (Table 3), thus reaching a BP cloning success rate of 93 % (Table 3).

**Table 3:** Success rates of ORF cloning in dependence on the template used

		**PCR**		**BP reaction**		**Entry clones**		
**ORFs targeted**	**# total**	**# pos**^a^	**%**	**# pos**^b^	**%**	**# +/- stop codon**^c^	**# + stop codon**^d^	**# - stop codon**^e^

**cDNA clones**	1712	1571	92	1477	94	1355	63	55
**EST clones**	368	341	93	314	92	290	9	13
**Primary cDNA**	156	85	54	76	89	69	1	3
**Sum**	2236	1997	89^f^	1867	93^g^	1714	73	71

^a ^Number of ORFs with expected product size obtained by PCR.

^b ^Number of ORFs with expected product size detected by colony-PCR of entry clones.

^c ^ORFs represented by at least one sequence verified entry clone with and without a stop codon.

^d,e ^At least one sequence verified entry clone with^(d) ^or without^(e) ^a stop codon was obtained for these ORFs.

^f,g ^Cumulative success rates of PCR reaction (^f^) and BP cloning (^g^)

### Simultaneous generation of ORF clones with and without a stop codon

**Figure 6:**
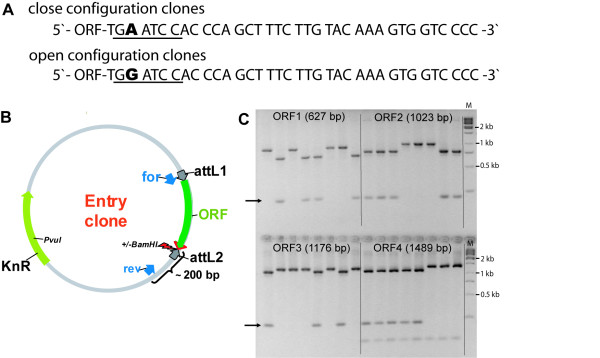
**Cloning strategy for the simultaneous generation of entry clones in open and closed configuration**. **A**: Sequences of entry clones 3' of the ORF either containing or not containing a stop codon. The sequences correspond to the reverse primer sequences of 2-step PCR. In presence of an A at the degenerated position, a stop codon is created and the *Bam*HI site (underlined) destroyed. In contrast, the inclusion of a G generates a *Bam*HI site and results in a translational read-through. **B**: Schematic presentation of the entry clone map. 'for' and 'rev' indicate the binding sites of the colony PCR primers. The degenerated position is indicated by the arrow. **C**: *Bam*HI colony-PCR restriction digest of eight independent colonies resulting from BP cloning of four different ORFs amplified using degenerated reverse primers. The arrows mark the additional band which appears in presence of the *Bam*HI recognition sequence, indicating that the ORF does not contain a stop codon. ORF 4 contains an internal BamHI site indicated by the appearance of a band of about 100 bp. 'M' indicates the molecular weight marker lanes.

Depending on the downstream applications the native stop codon of an ORF is required to be present or to be omitted in the cloning process, to allow for N- and/or C-terminal fusions, respectively. Localization studies employing fusion proteins with GFP have shown that proteins harbouring N-terminal leader sequences frequently mislocalize when they contain an N-terminal extension [10,38]. In contrast, expression of native proteins e.g. for protein structure determination [11,12] requires the presence of the native stop codon. To circumvent the laborious and cost-intensive duplication of all processes during clone generation and quality control, we developed a cloning strategy that allows for the simultaneous generation of two variants of Gateway entry clones, one containing a particular ORF with and the other without the stop codon. This was achieved by exchanging the native stop codon with a degenerated triplet (TGR) at the 3'-end of the ORF-specific sequence in the reverse PCR primer sequence leading to the inclusion of an A- or G-residue at the degenerated position during the PCR (Fig. 6a). The incorporation of an A-residue results in amplicons that contain a stop codon (TGA), the inclusion of a G changes it into a sense codon (TGG) for tryptophan. The primer design included the combination of that terminal triplet (TGR) with additional three bases resulting in a *Bam*HI recognition sequence only if the G was present, whereas the inclusion of the A destroyed the *Bam*HI site (Fig. 6a). For the nested PCR performed with a universal primer pair complementary exclusively to the overhangs (including the degenerated triplet) common to all first step primers, reverse primers containing either an A or a G at the degenerated position were purchased separately. They were mixed in a ratio of 1:1 to guarantee an equimolar ratio of the two primers in the tailed PCR.

For colony-PCR after *E.coli *transformation the nested PCR forward primer was used in combination with a reverse primer designed to anneal 200 bp downstream of the ORF to the vector backbone (Fig. 6b). PCR products were digested with *Bam*HI and the absence or presence of the stop codon was determined by agarose gel electrophoresis to distinguish the two species of entry clones. Clones with an open configuration displayed an additional band of 200 bp and a corresponding size shift of the ORF band in contrast to undigested clones containing a stop codon, as shown in Fig. 6c.

In summary, with this straightforward cloning protocol entry clones containing specific ORFs with and without a stop codon were obtained in parallel, while introducing only one additional working step, namely the *Bam*HI digest of colony PCR products. The success rate was > 90 % when eight individual entry clones were analyzed for every ORF. In few cases (< 5 %) only one of the two variants were found or no ORF (< 5 %) was present in the clones. Thus, the modification of the ORF flanking region in the 3'-primer did not significantly influence the recombination efficiency of the BP reaction. This strategy has a high capacity for automation and can thus be applied in high-throughput. It enabled the distinction of clones already before entry clone sequencing, saving the laborious and costly sequencing of randomly selected clones that would otherwise be required to identify ORF clones with as well as without a stop codon.

### Sequence validation of entry clones

Four entry clones per ORF scored positive by colony-PCR, two containing and two lacking a stop codon, were subjected to 5' and 3' sequencing using vector primers. The sequences were analysed for matching the target gene and for the integrity of the recombination sites to exclude clones containing primer or recombination errors. If the clones matched the target sequences the inserts were verified by complete sequencing using ORF specific primers. Entry clones were scored positive if the assembled sequences were identical to the expected sequences or if they contained base changes that were silent mutations or confirmed as SNPs. When base changes were observed that did result in amino acid substitutions they were evaluated as follows: If an alternative entry clones was present containing the correct ORF this clone was further used. Where amino acid substitutions were detected at different positions in the clones analysed, further clones were subjected to the sequencing process. If all clones contained the same amino acid substitutions cloning was repeated using an alternative template. Clones containing either nonsense mutations leading to in-frame stop codons or base changes within the recombination sites which potentially impaired the subcloning efficiency, were rejected. In cases where the ORF was not present or only partially cloned due to internal deletions or mispriming events or where introns were retained, the cloning was repeated. If the sequencing reaction failed new primers were designed.

Thus far, more than 3,800 entry clones have been sequence verified encoding more than 1,850 different ORFs (Table 4), mostly represented by at least one entry clone with and one without a stop codon (Table 3). The entry clone sequences are constantly submitted to the GenBank database. The improvements of particular cloning steps cumulated to our optimized cloning pipeline thus generating a clone collection which covers > 83 % of the targeted ORFs (Table 4) thereby an efficiency > 90 was reached for ORFs up to 2 kb. The clones are distributed via the ORFeome Collaboration [25] and are made available through I.M.A.G.E. clone providers.

**Table 4:** Overview on sequence validated accepted clones

	**genes**	**additional splice variants of the targeted genes**	**full-ORFs**^a^
**# initially targeted**	2021	215	2236
**sequence validated clones generated for**^b^	1681	177	1858
**% success**	83	82	83

^a ^Sum of ORFs encoded by targeted genes and splice variants.

^b ^At least one sequence verified entry clone with and/or without a stop codon was obtained

### Database application for sample tracking, standardization and quality control

**Figure 7:**
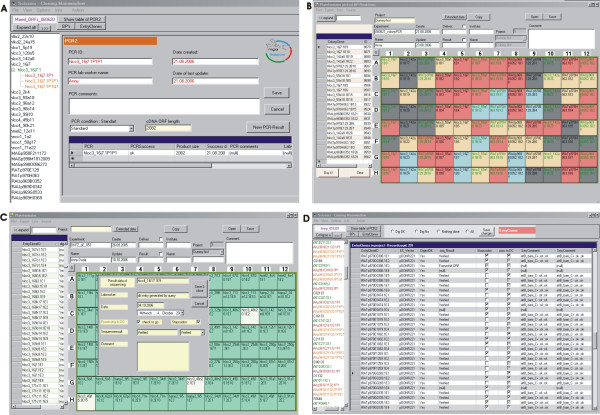
**User interfaces of the cloning database "SCISSORS"**. A: Screenshot of the data entry sheet of second step ORF PCR. B: 96-well colony PCR plate assembled by the software. The entered PCR results are automatically color-coded by the software as follows: red and grey: positive or negative colony (presence or absence of a band of expected size on the agarose gel), blue: entry clone colonies already used for plasmid preparation, yellow: colonies selected for generation of a new entry clone 96-well plate. C: User interface of entry clone plates. Clones scored positive in the control digest are automatically color-coded in green, negative clones remain white. Clicking on the plate positions opens a window to enter the sequencing result of the particular entry clone. D: Results of a working step can also be entered in a table format, as shown for the entry clone validation.

A database application has been developed as a prerequisite for the management and monitoring of a high number of ORFs in parallel and for the tracking of individual products in the cloning process. The software has been designed to automatically generate and maintain a standardized nomenclature during all steps of the cloning process, utilizing unique and consistent identifiers for ORFs, PCR reactions, colony-PCRs, entry- and expression clones (Fig. 7). Thus, possible errors and inconsistencies else likely introduced by manual typing could be avoided. New IDs are generated by the database and subsequent cloning steps are only enabled after the previous step has been quality controlled and approved to having been successful (Fig. 7a and 7c). All IDs contain a reference to the ORF models that were named according to the template used: in case of cDNA or EST clones according to the clone name and in case of primary cDNA according to the ORF prediction. Thus, the database implementation guarantees quality standards in the ORF-clone resources and a full tracking of each product. Working lists for the various experimental processes can be extracted to follow up on every particular ORF during the procedure (Fig. 7d). As all cloning steps have been performed in 96-well format (PCR, colony-PCR, entry clone preparation and sequence validation, and downstream processing of the entry clones), the respective plates could be automatically assembled by the database application (Fig. 7b and 7c). This greatly facilitated the automation of the cloning procedure, as the pipetting sheets could be directly transferred to the robotic workstations. In consequence, this contributed significantly to a streamlined cloning procedure and increased the cloning throughput and success rate. Furthermore, any functional data that are obtained with the ORF clone resource [13,14,23,24,39] and the corresponding expression constructs can be unambiguously tracked back to entry clones and the material they had derived from.

## Conclusion

We have described the ORF cloning pipeline of the German cDNA Consortium, where human full-length ORFs are manually modelled and annotated, and subsequently efficiently amplified and cloned into Gateway entry vectors. We have improved and streamlined protocols to circumvent possible size bias, to simultaneously generate ORF constructs with and without stop codons, and to automate most of the processes. SOPs describing the ORF cloning processes in detail are available at [33]. The German cDNA Consortium ORFeome resource currently consists of more than 3,800 sequence-verified entry clones for more than 1,850 ORF models, most of them cloned with and without a stop codon. These entry clones represent about 1,700 genes, 177 splice variants were cloned representing 121 of these genes. The entry clones allow for a broad range of subsequent applications to functionally characterize the ORF encoded proteins in multiple expression systems in parallel [1,13,14,23,24]. With this resource we significantly contribute to the international ORFeome collaboration [20] that aims at the generation and provision of a whole genome ORFeome collection of Gateway entry clones. The sequences are available at EMBL/GenBank/DDBJ databases and the clones are distributed via the ORFeome Collaboration and are made available through I.M.A.G.E. clone providers.

## Methods

### Gene annotation and modeling of new gene structures

Using the UCSC genome browser [40] for visualization, gene models were built based on mRNA, EST and gene prediction data. The HUSAR software package [41] was employed with its BLAST and ORF-prediction tools mostly for fine analysis and mapping of the gene structures, and to retrieve data from RefSeq [42] and EntrezGene [43] databases. The UCSC Table Browser function [44] was used to retrieve relevant sequences for subsequent joining to construct full-length ORF models for the different gene loci. Gene features rendered most relevant for full-length ORF selection were: EST- and mRNA coverage, presence of CpG islands, polyA signals, canonical splice signals, conservation from comparative genome data, exclusion of repetitive elements, and not to be target of nonsense mediated decay (NMD) [27,28]. If functional alternative splicing was observed for a gene locus different ORF models were build which were used as reference sequences for the generation of ORF cloning and sequencing primers for entry clone sequence verification. For ORF cloning we selected promising cDNAs or 5'-EST clones using our DKFZ or the MGC clone resources obtained via the RZPD (German Resource Center for Genome Research, Heidelberg). 5'-EST clones were first sequenced completely to analyze their potential to contain the full ORF. If no cDNA clones were available, suitable RNA sources were employed for RT-PCR to amplify full-length ORFs for subsequent cloning.

### ORF amplification by PCR

The amplification of ORFs had originally been performed in a single PCR reaction as described previously [10], and has since been replaced by a 2-step procedure [30] performed on 96-well format. Primers for first-step PCR were designed using the PRIDE program [45] and purchased salt free from three different suppliers. The standard PCR contained a final concentration of 1x Phusion HF buffer, 10 ng template DNA, 10 pmol of primers, 0.5 mM dNTPs and 0.5 U Phusion DNA polymerase in a total reaction volume of 25 μl. Standard first-step PCR parameters were: 98°C for 30 sec, 12 cycles of 98°C for 10 sec, 55°C for 10 sec, 63°C for 15–30 sec/1 kb, 63°C for 5 min final extension. The Gateway™ recombination sites were completed in a second PCR using a universal pair of PAGE-purified primers (Eurogentec). Forward primer: GGGGACAAGTTTGTACAAAAAAGCAGGCTCCACCATG; reverse primer: GGGGACCACTTTGTACAAGAAAGCTGGGTG (underlined sequences overlap with primers of first-step PCR). The nested PCR was performed in a 50 μl reaction volume consisting of 1–5 μl of first PCR reaction, 10 pmol of primers, 1 mM dNTPs, 1x Phusion HF buffer and 1 U Phusion DNA polymerase. The standard cycling conditions were identical to those of the first-step PCRs. For PCR product purification ethanol precipitation as well as other methods including QIAquick PCR Purification (Qiagen), ChargeSwitch PCR Clean-Up (Invitrogen), QIAquick Gel extraction (Qiagen) or MinElute Gel Extraction (Qiagen) were compared for best results. Detailed protocols for the two-step ORF amplification process are available at [33].

### BP cloning of PCR-products

PCR products were cloned by BP recombination (Invitrogen) [22] into pDONR201 or pDONR221 in 96-well format according to the supplier's instructions, except using only half of the recommended volumes [22]. Incubation was at 25°C for 2–20 h. Ca^2+^-competent DH10B *E.coli *bacteria were transformed with the BP product using a Multiprobe pipetting robot (Perkin Elmer). Transformants were spread in two Q-trays (22 × 22 cm, Genetix), each subdivided into 48 squares by plastic grids, and containing LB agar supplemented with 50 μg/ml kanamycin. Eight colonies per ORF were analysed for the presence of the ORF of expected size in a colony PCR, utilizing the Perkin Elmer Multiprobe robot to set-up the reactions. Simultaneously, the colonies were inoculated into a 96 deep well block (Greiner) and bacteria were grown for 16 hours.

### Generation of ORF clones in open and closed configuration

ORFs both with and without a stop codon were generated simultaneously by introducing the following protocol modifications: six additional base pairs (underlined in the primer sequences below) were added upstream of the ORF-specific sequence in the reverse PCR primer for the first PCR step. One of these base pairs represented a degenerated position (Y = C or T): 5'-TGGGTGGATYCA-ORF-specific sequence-3'. For the nested PCR two reverse primers were mixed in an equimolar ratio, each containing either a "C" or "T" at the degenerated base position of the first step primer. For entry clone analysis by colony-PCR the second step ORF-PCR forward primer was combined with the following reverse primer: 5'-TCTTGTGCAATGTAACATCAG-3'. Subsequently, the reaction volume was doubled and 2 units of *Bam*HI were added directly into the wells of the 96-well colony PCR plate in order to screen for clones with and without a stop codon. After 2 h incubation at 37°C the samples were analysed on agarose gel.

### Sequence validation of entry clones

Four entry clones of every ORF two with and two without a stop codon that had been scored positive in the colony PCR (Fig. 6c) were rearrayed using the Mulitprobe pipetting robot. Plasmid preparation was done with the Nucleospin Robot-96 plasmid kit (Macherey-Nagel) on the Bio Robot 9600. Entry clones were subsequently monitored by *Bsr*GI single and *Bam*HI/*Pvu*I double digest. Clones scoring positive were subjected to automated sequencing on 3100 Genetic Analyzers (Applied Biosystems) with BigDye Terminators v3.1 (Applied Biosystems). The entry clones were completely sequence-verified including the Gateway recombination sites applying primer walking strategy. The primer were designed to aneal every 450 bp based on the reference sequence of the ORF model using the PRIDE program [45]. Sequences were assembled using the Staden package [46] together with the reference ORF model sequence and checked for differences. Entry clones sequences were annotated based on the reference sequences using the Blast tools of the HUSAR software package [41]. Sequences are constantly submitted to the GenBank database.

### Cloning database

The software for cloning process management ("SCISSORS") is a MS .NET application using MS SQL Server as a database backend. The software is a Lab Information Management System (LIMS) providing user interfaces for working step management, data acquisition and analysis. It furthermore represents an administration tool for clone and plate storage and is also used to store and display clone annotation information.

## Authors' contributions

ORF amplification and cloning, process optimization and improvement, entry clone sequencing and development of the cloning strategy for the simultaneous generation of ORFs in open and closed configuration was done by SB. IS has been involved in gene structure modeling and in the selection of full-length cDNA clones, the automated ORF primer design, and entry clone sequence validation. HR and AM developed the process management software. Cloning and analysis step automation was done by CPS. AD, UE, AB, HB, DH, AH, GM, HW, KK and BO did the experimental work in cloning and sequencing. RW participated in the DNA polymerase screening and CS assisted in editing the manuscript. SW participated in the Gateway cloning and in entry clone sequencing. SW and AP initiated the project, SW is coordinator of the cDNA Consortium. All authors read and approved the final manuscript.
